# Diagnosis and surgical decision-making of a 46, XX ovotesticular disorders of sex development patient: a case report

**DOI:** 10.3389/fsurg.2024.1502340

**Published:** 2024-12-11

**Authors:** Hanxing Zhao, Zhixing Chen, Baoyun Wang, Zhenyu Zhang, Zhengyong Li

**Affiliations:** ^1^ Department of Burn and Plastic Surgery, West China Hospital, Sichuan University, Chengdu, China; ^2^ Department of Plastic Reconstructive and Aesthetic Surgery, West China Tianfu Hospital, Sichuan University, Chengdu, China

**Keywords:** ovotesticular disorder of sex development, disorder of sex development, diagnostic process, feminizing genitoplasty, external genital abnormalities

## Abstract

**Background:**

Ovotesticular disorder of sex development is a rare form of disorder of sex development that manifests as ovotestis in individuals. The precise diagnosis and the choice of surgical procedures are still in conflict condition due to the rarity of the disease, diverse clinical presentations, and the lack of evidence-based medical studies on postoperative outcomes.

**Case presentation:**

We present a 46, XX ovotesticular disorder of sex development case, aged 19, with Prader stage IV virilization who underwent feminizing genitoplasty surgery. Our surgical strategy prioritized the patient's genitourinary function restoration and cosmetic reconstruction achieved satisfactory results. We attribute the success of the treatment to the systematic diagnostic process and individualized surgical planning.

**Conclusion:**

The purpose of this article is to provide an evaluation protocol for the ovotesticular disorder of sex development, improving the diagnostic rate and providing some fresh ideas for surgical management.

## Introduction

Ovotesticular disorder of sex development (OT-DSD) is a rare form of disorder of sex development that manifests as the presence of either an ipsilateral gonad with ovotestis or bilateral gonads with separate ovary and testis (1). The clinical phenotypes of 46, XX virilized patients are varying degrees of external genital virilization with internal genitalia hypoplasia and urinary malformation (2). The diverse clinical manifestations and complex urogenital malformations often lead to misdiagnosis and inadequate treatment of patients (3).

Considering progress in the clinical management of DSD has been made after the Chicago Consensus on the management of intersex disorders published in 2006 (2). Yet, only a few retrospective studies on the outcome of surgical treatment have been published in the last 20 years. Therefore, there needs to be more evidence-based data to support the surgical outcomes of OT-DSD. Here, we present the diagnostic process and personalized surgical management for a 19-year-old female OT-DSD patient, laying the foundation for promoting the development of a standardized treatment process for 46, XX OT-DSD. Informed consent was obtained from the patient and her guardians concerning the surgical plan, potential risks, alternative surgical strategies, and the publication of patient-identifying information.

### Case presentation

#### Chief complaints

A 19-year-old female presented to the outpatient department of plastic surgery with the chief complaint of genital ambiguity and requested a feminizing genitoplasty. She is raised as a female and has adopted a female gender identity.

#### History of present illness

The patient had genital ambiguity at birth and was considered male hypospadias by the physicians at that time. The patient was regularly followed up in the pediatric clinic during early childhood. At age 3, the patient had a karyotype analysis with a result of 46, XX. The female sex was subsequently assigned based on the results of karyotype analysis and the parents' preference. The patient remained untreated for the past 19 years.

#### History of past illness

The patient was in good health and had no other disease in the past.

#### Personal and family history

Breast development and menstruation started at the age of 15. The patient had regular menstrual periods every month with a normal menstrual flow associated with dysmenorrhea. The patient denied any family history of DSD. Her mother disclaimed having taken steroids in the early pregnancy stage.

#### Physical examination

**Figure 1:**
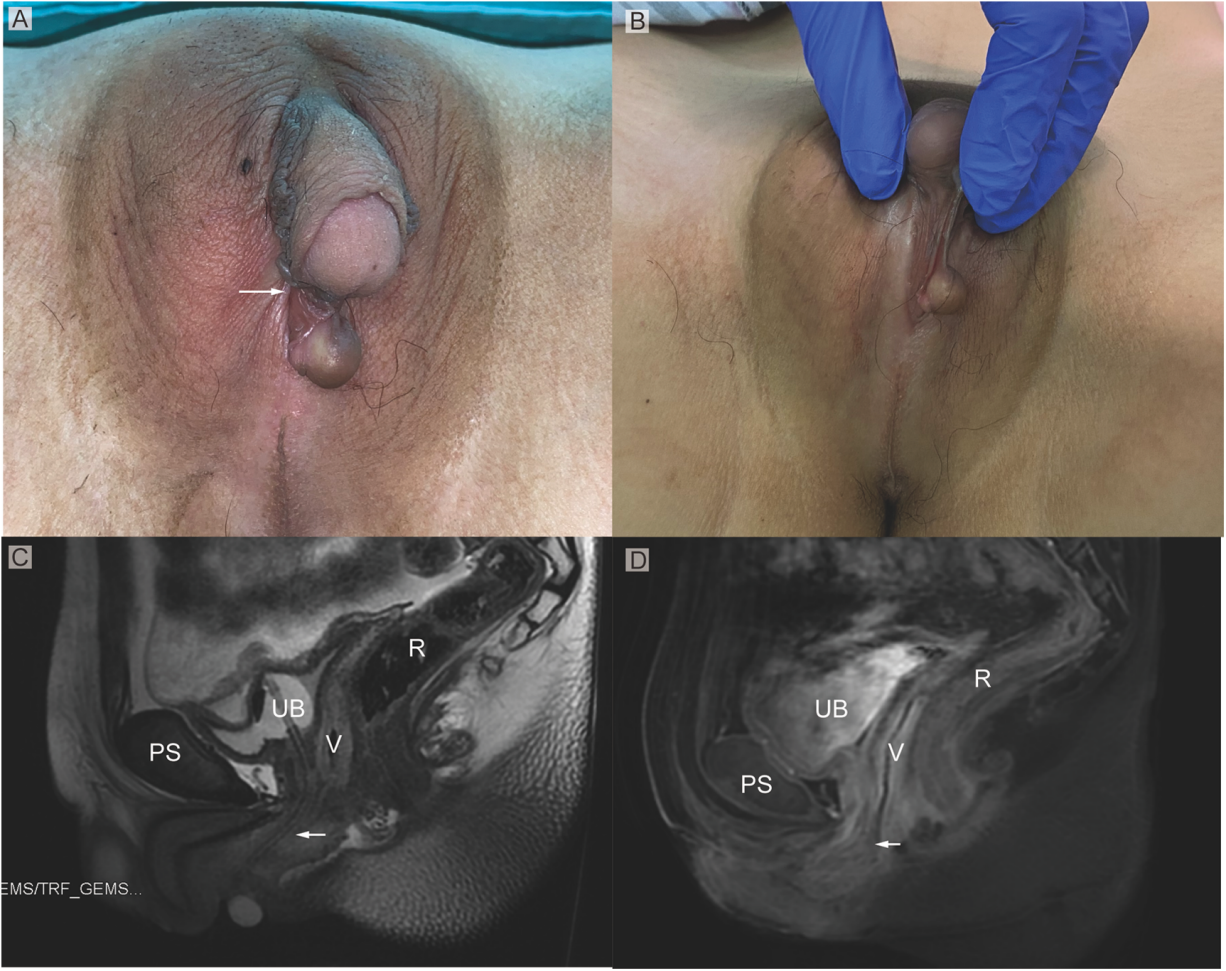
**(A)** Phenotypic appearance of genitalia before surgery. An enlarged clitoris, completely fused labia majora, and a movable painless mass at the right inguinal region was found. **(B)** An opening of the urogenital sinus was seen at the base of the clitoris. **(C,D)** Preoperative contrast-enhanced MRI showing well-defined urogenital sinus. Uterus was not detected. Sagittal MRI scan showing urinary bladder (UB), vagina (V), the common opening of the urogenital sinus (arrow), and rectum (R**)** The distance between the conference point to the superficial perineal pouch was below the level of the distal end of pubic symphysis (PS).

On physical examination, the vital signs were normal. The vulva showed Prader 4 virilization, presenting as penile-like clitoral hypertrophy, complete fusion of the labia majora, and an opening of the urogenital sinus at the base of the clitoris (Figures 1A,B). No signs of the presence of Bartholin's glands were found during the examination. The patient complained that menstruation and urine were drained from the orifice. The patient stated that has no history of erection. In the standing position, a movable, painless mass could be found in the right inguinal region, which disappeared in a resting position.

#### Laboratory examinations

Laboratory tests of E2 (17α-estradiol), LH (Luteinizing hormone), FSH (Follicle-stimulating hormone), PRL (Prolactin release hormone), AMH (anti-Müllerian hormone), hCG (human chorionic gonadotrophin), ACTH (Adrenocorticotropic hormone), testosterone, and serum electrolytes were within normal reference range. Routine blood and urine tests showed no abnormalities.

#### Imaging examinations

On MRI examination, a hypoplastic gonad was found in the pelvis (Figures 1C,D). A subcutaneous cystic mass was detected in the right labium. The distance between the conference point to the superficial perineal pouch was below the level of the distal end of pubic symphysis about 2 cm (Figures 1C,D). However, the uterus was not found on the radiological examination.

#### Further diagnostic workup

**Figure 2:**
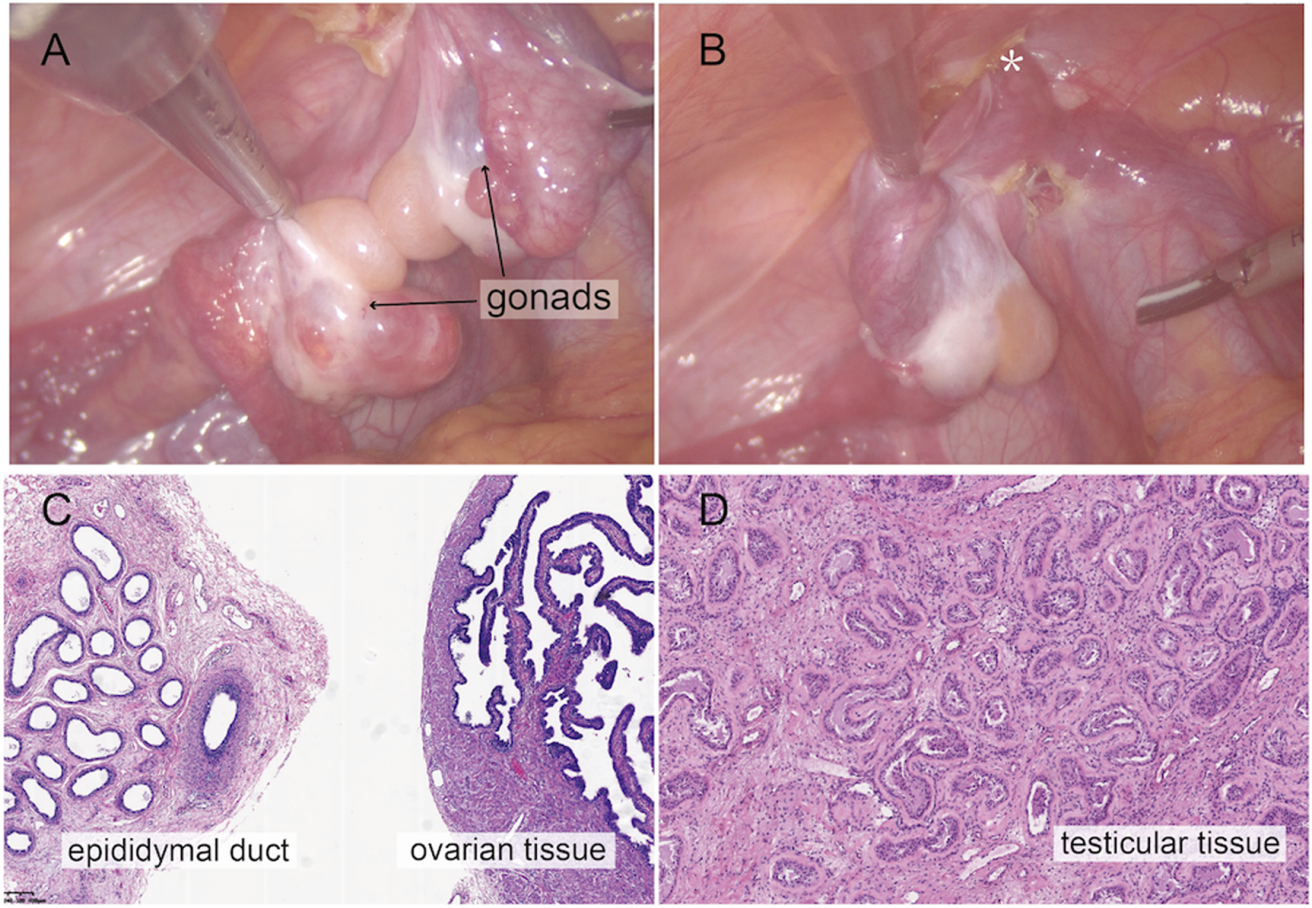
Intraoperative findings from laparoscopic exploration. **(A)** Intra-operative image of heterogenous gonadal structure showed the testicular and ovarian fractions organized in an end-to-end fashion, suggesting the ovotestis. **(B)** The right gonad was found at the inner ring of inguinal canal (*). **(C)** A biopsy was taken from the right hernial gonad in order to rule out any dysgenesis or ovotesticular tissue. Histopathological examination of sample 1 revealed epididymal and ovarian tissue. **(D)** Histopathological examination of sample 2 showed testicular histology with seminiferous tubules.

Urethroscopy further verified the MRI findings. Urethroscopy revealed a tubular urethral canal with a narrow vaginal introitus, 2 cm away from the skin, leading to a vagina. The introitus did not allow insertion of the endoscope into the vagina. A Foley catheter was placed into the vagina under urethroscopy to locate the confluence of the urethra and the vagina. A subsequent laparoscopic gonad biopsy was conducted. Intraoperatively, bilateral heterogeneous gonads were found (Figure 2A), with the right gonad located at the inner ring of the inguinal canal (Figure 2B) and the left gonad located in the pelvis. Histopathological examination of the right gonad confirmed that the sample contained epididymal, testicular, and ovarian tissue with no malignant tissue (Figures 2C,D).

#### Multidisciplinary expert consultation

The multidisciplinary (MDT) consultation, centered on diagnosis and surgical decision-making, was attended by specialists in plastic surgery, endocrinology, gynecology, urology, gynecology, psychology, and social department. According to the spectrum of evaluations, the diagnosis of 46, XX OT-DSD was confirmed. An open communication between the MDT group and the patient regarding gender identity, gender roles, and the patient's concerns was conducted before decision-making. Since the patient's gender identity was female, the MDT specialists agreed that the patient had an indication for female genitoplasty and required hormonal supplementation postoperatively.

#### Surgical treatment

**Figure 3:**
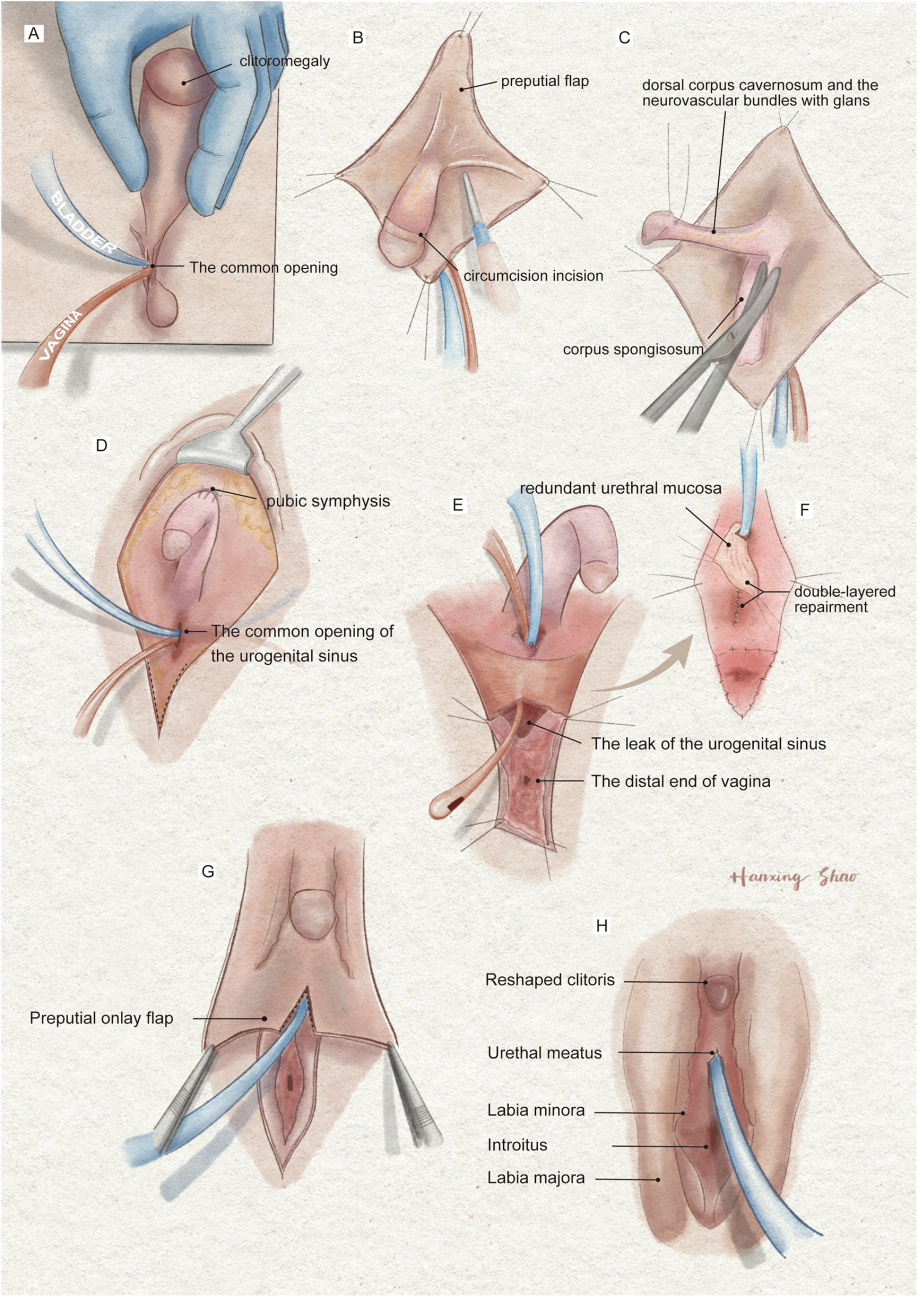
The illustration of the surgical procedures of feminizing genitoplasty. **(A)** The vulva showed Prader 4 virilization. **(B)** A subcoronal circumferential incision was made to de-glove the clitoris over Buck's fascia. **(C)** The corporal bodies were excised with preservation of the glans clitoris and neurovascular bundles. **(D)** The corpora cavernosa was folded and sutured to the pubic arch. **(E)** The orange urethral catheter entered from the orifice through the urogenital sinus to exit from the fistula. **(F)** The fistula was sutured and secured with the rotating urethral mucosa flap. The vagina was pull-down to the vulve and sutured to the skin. **(G)** The prepuce in an onlay fashion covers the area between the clitoris and the vestibule. **(H)** The final result of the procedure.

Individualized feminizing genitoplasty included clitoroplasty, vaginoplasty, and urogenital sinus repair, along with labiaplasty. Preoperatively, a Foley catheter and an orange urinary catheter were placed into the bladder and vagina, respectively, under urethroscopic guidance (Figure 3A). A reduction clitoroplasty was achieved through a ventral approach to the corporal bodies and subcoronal circumferential incision proximal to the glans clitoris (Figure 3B). The corporal bodies were excised with preservation of the glans clitoris and neurovascular bundles under the pubis (Figure 3C). The preserved clitoris, allocated for neoclitoris, was refined to about 1 cm size and recessed inferior to the pubic arch by securing sutures between the dorsal corporal facia and the inferior margin of the symphysis (Figure 3D). Then, vaginoplasty and urogenital sinus repair were performed. A longitudinal incision is performed regarding the vestibule where the neovagina and urethral meatus will be located (Figure 3E). During surgical dissection, we have observed the vestibule of the vagina, the labia minora, the labia majora, the vestibular bulbs, and the female corpus spongiosum were completely fused resemble a normal scrotum. Blunt dissection of the soft tissue and corpus spongiosum was carefully performed with the assistance of the Foley catheter to create space for the vagina. The distal end of the vagina was separated from the urethra at the confluence. Subsequently, the leak in the urinary tract was sutured and reinforced with excess urethral plate (Figure 3E). The urethral mucosa was rotating downward to cover the area between the urethral meatus and the vagina serving as a nonkeratinized vestibule. The tabularized neovagina was then created by pulling down and anastomosing the proximal vagina end with the skin and the redundant preputial flap (Figure 3F). Finally, the preputial flap, in an onlay fashion covering the area between the clitoris and the vestibule, was allocated for contouring of the labia majora (Figure 3G). The edges of the flap were sutured with vestibule medially and perineal skin laterally with interrupted 6-0 Vicryl (Figure 3H).

#### Outcome and follow-up

Surgical outcomes, including the size and position of the neo-clitoris, position of the urethra, dimension of the neovaginal cavity, size and symmetry of the labia minora and the labia majora, and ease of introitus, as well as surgical complications, were followed up. No complications except for introitus stenosis were found. Vaginal dilation and hormonal treatment with Femoston (estradiol 2 mg/progesterone 10 mg) were started 4 weeks after operation. Menstruation carried on 1 month after surgery with a duration of 5 days. The patient had not yet had sexual intercourse, but the vaginal meatus could be easily probed with a 5-cm-diameter dilator 6 months after surgery.

## Discussion

The main clinical manifestations of OT-DSD are virilized changes of the genitalia, with varying degrees of clitoral hypertrophy, labial fusion, and the presence of urogenital sinus and uterine hypoplasia. The vast majority of patients with 46, XX virilization may have congenital adrenal hyperplasia (CAH), but OT-DSD should be considered if there is a labial or inguinal gland, underdeveloped uterus, or the hormone levels suggestive of a normal adrenal steroid test (4). We have described the systematic diagnostic process of this patient to provide a reference for the future diagnosis and treatment of similar complex malformations.

**Figure 4:**
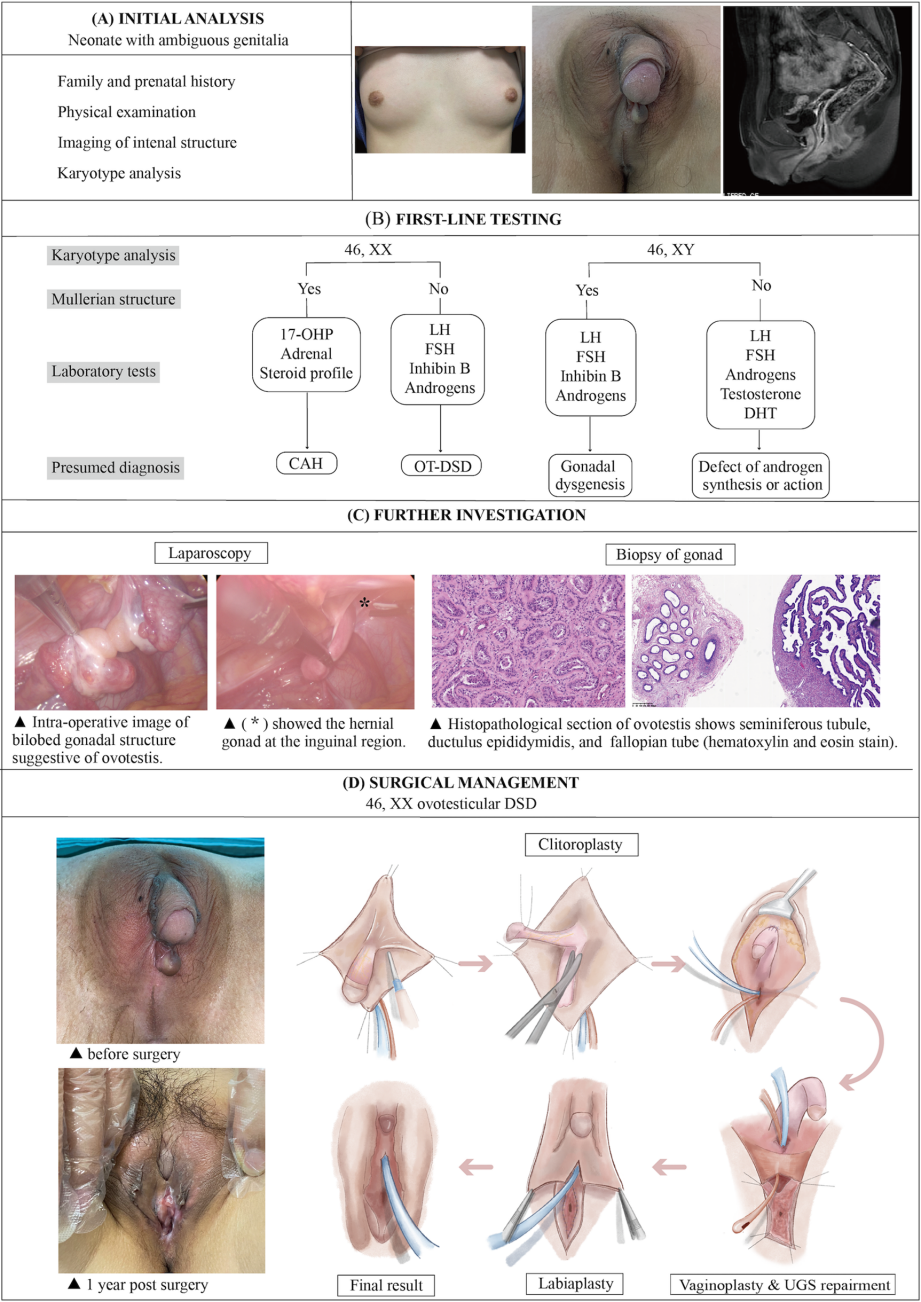
The illustration of the systematic diagnostic and treatment process for OT-DSD. The investigation process of OT-DSD can be summarized as initial assessment **(A)**, first-line testing **(B)**, and further investigation **(C,D)** showed the procedures of feminizing genitoplasty and pre- and 1-year post-operative pictures. The procedures included clitoroplasty, vaginoplasty and urogenital sinus repair, along with labiaplasty. 17, OHP, 17-alpha hydroxy progesterone; LH, Luteinizing hormone; FSH, follicle-stimulating hormone; DHT, dihydrotestosterone; CAH, congenital adrenal hyperplasia; OT-DSD, ovotesticular disorder of sex development.

The diagnostic plan and the surgical procedures of OT-DSD were summarized in three stages: initial assessment, first-line testing, and further investigation (Figure 4). For reasons such as the psychosocial development of the child, the current consensus favors assigning gender to all patients after completion of the initial analysis, after careful consideration of the degree of external virilization, nature and function of gonads, uterine development, karyotypes, and parental predilection (4). The final decision on assigning sex of rearing is organized in a family-centered multidisciplinary setting, including pediatric endocrinologists, urologists, plastic surgeons, gynecologists, geneticists, psychiatrists, and social workers (5).

To better ensure the physical and emotional growth and development of patients with disorders of sex development the European Society for Pediatric Urology stated that the treatment is best organized by the multidisciplinary team with the patients and their family (6)*.* There are few long-term follow-up reports on the frequency of gender dysphoria in patients with OT-DSD. Previously, one male-assigned patient with OT-DSD (1/23, 4.3%) showed sex dysphoria after a unilateral gonadectomy and was reassigned as female at the age of 20 (7). In contrast, a 40-year follow-up study did not find gender issues in 33 cases with OT-DSD after conservative gonadal surgery (8). Assigning sex of rearing at an early stage in accordance with following up steroid replacement and surgical intervention were recommended by the Fourth World Congress of the International Society of Hypospadias and Disorders of Sex Development Surgery and the American Academy of Pediatrics which can reduce the stigma associated with genital atypicality (9, 10).

The hCG stimulation test can define the existence of functional testicular tissue in adults. However, these results need to be interpreted in the context of patient's age and pubertal stage (5). The consensus suggested removing the testes in female-assigned patients to avoid malignancy in adulthood (11). However, OT-DSD patients have a low risk of malignancy (2.6%–4.6%) and can leave the testes in place (12). In our case, this is exactly how we preserved the ovotestis in the pelvis to preserve partial ovarian function. Current recommendations are long-term follow-up at puberty seeking signs of gonadal malignancies for female-assigned OT-DSD patients.

The definitive diagnosis is based on either an ipsilateral gonad with ovotestis or bilateral gonads with separate ovary and testis. Laparoscopic evaluation of internal genital structures, including gonadal biopsy, is performed to finalize the diagnostic process. However, in those patients who undergo gonad biopsy without finding ovotestis, it may result in misdiagnosis.

In recent years, the promotion of genetic testing has contributed to the investigation of the genetic background of OT-DSD. The Y chromosome sex-determining region (SRY) gene triggers testicular differentiation from early bipotential gonads by up-regulating SRY-box transcription factor 9 (SOX9) and steroidogenic factor 1 (SF1) (13, 14). The development of ovotestis has been shown to be associated with gain-of-function changes in male sex-determining genes or their regulatory regions or loss-of-function mutations in female sex-determining genes (15–17). Therefore, genetic testing for genes related to OT-DSD can prevent misdiagnosis.

The goals of genitoplasty are a normal genital appearance with a sensate clitoris and a suitably sized vagina to make the vagina suitable for menstruation and penetrative intercourse, while also reduce urologic problems, reduce the risk of gonadal cancer and facilitating the psychosocial development of patients.

The embryological development of the genitalia starts at 9 weeks of gestation and complete by 12 weeks of gestation (18). The origins of the external genitalia include the genital tubercle, the urethral folds, and the labioscrotal swelling. The female genital organs differentiate in the male genital organ development during the embryonic period without androgenic control that the genital tubercle forms the clitoris and the labioscrotal folds form the labia major and minora and lower vagina (19). The urethral meatus is located at the tip of the vulva in female, just above the vaginal introitus. The vestibule is bordered anteriorly by the clitoris, posteriorly by the perineum. They are normally at a deeper plane bordered by the labia minora. DSD result from problems with the chromosomal, gonadal differentiation, and hormone production or response.

Vaginoplasty and urogenital sinus repair are vital steps in feminizing genitoplasty, as this may result in urinary fistulae, vaginal stenosis, urinary incontinence, or early suture dehiscence (20). The length of the UGS and the specific location of the vaginal confluence have directed the choice of procedure. In this case, the preoperative evaluation suggested an adequate length of the remnant vagina. Therefore, the pull-down procedure allows the vaginal confluence easily reach the perineum. After separate the urethra from the vagina, we used the redundant urethral mucosa to reinforce the fistula which avoid the urinary fistulae.

Other grafts reported in the literature for vaginoplasty include buccal mucosa and the large intestine, sigmoid colon, and the penoscrotal flap. There was concern regarding the possibility of vaginal stenosis, graft necrosis, urethral stenosis, and rectovaginal fistula after these more “aggressive” procedures (21–23). Hennayake S et al. in 2022, reviewing all morphological observation of the vaginal introitus and its relationship to the perineal body concluded that not all patients need local flaps or mobilisation of the uroginital sinus. For those vaginal introitus were observed below the level of the distal end of pubic symphysis, the peripheral tissues can be moved up to the vagina (24).

The 2-year follow-up result showed that this technique is feasible and effective in achieving female appearance genitalia. There are several advantages to our feminizing genitoplasty. The advantages of the technique include: (1) Our surgical approach fully used adjacent tissues to avoid damage to the donor area. (2) The prepuce of the enlarged clitoris provided a hairless lining for labia minora reconstruction. (3) The simplicity of the technique, with no need to harvest grafts from other sites, controls the risks of the procedures.

Due to the diverse clinical presentations, the spectrum of findings, and diagnoses, we do not believe our evaluation protocol can apply to all cases. However, initial analysis and first-line tests, including family and prenatal history, physical examination, karyotype analysis, hormone tests, and imaging, are essential in diagnosis and treatment. For patients with disorders of sex development, the development of an evaluation protocol can help in making an accurate diagnosis. Although the current case used the residual vagina for vaginoplasty, due to the patient's underdeveloped reproductive system, the patient experienced vaginal stenosis postoperatively and will need further surgery or vaginal dilation in the future. Another limitation of the study involves the measurement of the depth of the remnant vagina. Since the crypt did not allow insertion of the endoscope into the vagina, the depth of the vagina was measured by MRI imaging to indicate the choice of surgical approach for vaginoplasty. A remaining limitation is the relatively small sample size and the follow-up lasting only 2 years. In the future, we will expand the sample size and have a longer follow-up period.

## Conclusion

Ovotesticular disorder of sex development is a rare form of disorder of sex development with varying degrees of external genital virilization with internal genitalia hypoplasia. In this report, we present a 46, XX OT-DSD case with severe virilization who underwent minimally invasive feminizing genitoplasty surgery. The patient was satisfied with the appearance of the external genitalia and underwent regular vaginal dilatation without any complications during the long-term follow-up. In conclusion, systematic diagnostic procedures can reduce the incidence of misdiagnosis. Our individualized feminizing genitoplasty has controlled the occurrence of postoperative complications and obtained good surgical outcomes.

## Data Availability

The original contributions presented in the study are included in the article/Supplementary Material, further inquiries can be directed to the corresponding authors.
